# The strategy of composite grafting with BMP2-Loaded calcium phosphate cements and autogenous bone for alveolar cleft reconstruction

**DOI:** 10.3389/fphys.2022.1023772

**Published:** 2022-09-27

**Authors:** Hongzhou Shen, Lvyuan Li, Chenglong Zhang, Yang Chen, Hongbo Yu, Jiawen Si, Guofang Shen

**Affiliations:** ^1^ Department of Oral and Craniomaxillofacial Surgery, Shanghai Ninth People’s Hospital, College of Stomatology, Shanghai Jiao Tong University School of Medicine, Shanghai, China; ^2^ Department of Orthodontics, Shanghai Ninth People’s Hospital, Collage of Stomatology, Shanghai Jiao Tong University School of Medicine, Shanghai, China

**Keywords:** bone morphogenetic protein 2, calcium phosphate cements, bone regeneration, bone graft, alveolar bone grafting

## Abstract

**Purpose:** To remedy the drawbacks of traditional autogenous bone harvesting in alveolar bone grafting (ABG), a novel strategy of composite grafting with BMP2-loaded calcium phosphate cements (BMP2-CPC) and autogenous bone harvested by minimally invasive technique was developed and evaluated for its bone-repairing efficacy.

**Materials and methods:** A chart review was conducted for 19 patients with unilateral alveolar clefts who underwent secondary ABG from 2017 to 2020. Of the enrolled patients, 9 patients underwent grafting with autogenous bone harvested by traditional trap door technique (group I), and 10 patients underwent grafting with the composite graft comprising BMP2-CPC and autogenous bone harvested by minimally invasive technique at a ratio of 1:1 by volume (group II). The clinical performance of the composite graft was comprehensively evaluated in terms of clinical, radiographic and histological perspectives.

**Results:** The present results demonstrated that the composite graft exhibited satisfactory bone-repairing efficacy comparable to that of the autogenous bone graft on the premise of lower amount of harvested bone. The post-surgical resorption of bone volume and vertical height of grafted area was significantly slower in group II. The favourable resorption performance of BMP2-CPC contributed to preserving the post-surgical bony contour reconstructed with the composite graft.

**Conclusion:** The composite graft comprising BMP2-CPC and autogenous bone harvested by minimally invasive technique was demonstrated to be an eligible alternative for application in ABG, especially for its improved resorption performance in preserving post-surgical bony contour.

## Introduction

Currently, cleft lip, alveolus and palate, with a high incidence ratio of 1:700 live births, are the most common human congenital craniofacial anomalies (Guo et al., 2011). Among the multidisciplinary sequential treatments for lip-alveolus-palate cleft patients, secondary alveolar bone grafting is a fundamental step for the stabilization of the maxillary dental arch, facilitation of prosthodontic and orthodontic treatment, induction of canine eruption, closure of vestibular fistulae and improvement of nasal symmetry and facial morphology (Guo et al., 2011; Chhajlani et al., 2021; Francisco et al., 2021). Although autogenous bone graft has still been suggested as the “gold standard” for ABG, its drawbacks, such as donor site morbidity, limited bone supply and post-surgical resorption, are also prominent (McCrary and Skirko, 2021; Dissaux et al., 2022).

Facing this dilemma, clinicians and researchers have developed various grafting strategies to remedy the drawbacks of autogenous bone graft. Previous reviews of clinical trials identified several potential alternatives for ABG: BMP2, deproteinized bovine bone (DBB) and synthetic grafting materials [hydroxyapatite (HA), β-tricalcium phosphate (β-TCP), calcium phosphate cements (CPC)] (Liang et al., 2018; Dissaux et al., 2022). Among these materials, BMP2 was intensively studied and showed good bone-repairing efficacy comparable to that of autogenous bone graft in terms of reconstructed bone volume and density (Osorio et al., 2020; Francisco et al., 2021; Alkaabi et al., 2022). However, the current carrier for BMP2, absorbable collagen sponge (ACS), exhibits poor structural stability and possesses no controlled-release capability, which cannot fully meet the clinical requirements of ABG (5, 9). As another common alternative to autogenous bone graft, DBB was recommended by clinicians with the purpose of reducing surgical trauma and shortening post-surgical healing period (Thuaksuban et al., 2010; Aly and Hammouda, 2016; Bezerra et al., 2019). Although previous clinical radiographic evidence indicated that the use of DBB alone or combined with BMP2 was as successful as autogenous bone in repairing alveolar cleft (Thuaksuban et al., 2010; Francis et al., 2013; Aly and Hammouda, 2016; Sharif et al., 2016; Liang et al., 2017; Bezerra et al., 2019), such finding still needs to be validated by histological examination. Moreover, the inherent drawbacks of DBB, potential disease transmission, poor osteoinductivity and slow resorption rate significantly limit its clinical application in ABG (Sharif et al., 2016; Kang, 2017; McCrary and Skirko, 2021). Therefore, synthetic grafting materials with favourable biocompatibility, osteoconductivity, osteoinductivity and adjustable physical and mechanical properties were in urgent need for the treatment of alveolar cleft (Kamal et al., 2018; Osorio et al., 2020).

Previously, Lin et al. (Lin et al., 2016) reported a novel BMP2-CPC material resulting in a shortened bone-repairing period and improved post-surgical bone quality when applied in the clinical treatment of long bone fractures. Compared to DBB, BMP2-CPC exhibited stronger osteogenic capability and better resorption performance in the setting of guided bone regeneration (Shen et al., 2021a). Moreover, BMP2-CPC facilitated an improved immune response pattern characterized by up-regulated M2-phenotype polarization in macrophages, alleviating local inflammatory reactions and enhancing bone regeneration at defect sites (Shen et al., 2021b). Based on this evidence, we propose BMP2-CPC as an alternative to autogenous bone graft for treating alveolar cleft due to its favourable biological properties and potent osteogenic capability.

However, the bone-repairing efficacy of BMP2-CPC was confirmed only in the treatment of long bone fractures (Lin et al., 2016), while relevant studies evaluating BMP2-CPC in repairing alveolar cleft are still lacking. In this study, we adopted a strategy of composite grafting with BMP2-CPC and autogenous bone to evaluate the application of BMP2-CPC in the alveolar cleft reconstruction. The clinical performance of BMP2-CPC was comprehensively evaluated in terms of clinical, radiographic and histological perspectives. Through this study, we hope to establish a new possibility to bridge the alveolar cleft on the premise of effective bone regeneration and minimized surgical trauma.

## Materials and methods

### Grafting material preparation

The grafting material, BMP2-CPC (Rebone^TM^, China), used in this study was purchased from Rebone Biomaterials Co., Ltd. (Shanghai, China). Briefly, the porous CPC scaffold was prepared through salt leaching and molding method. CPC scaffold was cast into the mold under a pressure of 2 MPa for 1 min. After 3 days of curing (37°C and 100% air humidity), CPC was soaked in purified water for 3 days to remove the NaCl particles and dried at 100°C for 12 h. After sterilization of CPC scaffold, BMP2-CPC was prepared by dropping BMP2 protein onto CPC scaffold and evacuating for 30 min to entrap BMP2 into the porous structures of CPC scaffold. The loaded amount of BMP2 was 1 mg/g (mg BMP2 protein/g CPC scaffold).

### Study design

This study was conducted in accordance with the principles of Declaration of Helsinki (2013) and approved by the Independent Ethics Committee of Shanghai Ninth People’s Hospital affiliated with Shanghai Jiaotong University, School of Medicine (SH9H-2021-T176-1). The clinical application of BMP2-CPC was approved by China Food and Drug Administration (CFDA Certified No. (2013): 34–60199). A review of patient charts and radiographs was performed for all patients who underwent secondary ABG surgery at the Department of Oral and Craniomaxillofacial Surgery, Shanghai Ninth People’s Hospital from January 2017 to December 2020. Nineteen patients (13 males and 6 females) with unilateral alveolar cleft were finally enrolled in this study. Surgical and orthodontic notes were reviewed to identify occurrences of post-surgical complications (infection, oronasal fistula, graft failure, etc.) and evaluate canine eruption.

The inclusion criteria were as follows: 1) patient was 8–15 years of age; 2) patient had unilateral alveolar cleft; 3) autogenous bone was harvested from the anterior iliac crest; and 4) all surgeries were performed by the same senior maxillofacial surgeon. The exclusion criteria were as follows: 1) incomplete clinical data; 2) history of periodontal disease; 3) history of diabetes mellitus; 4) history of alcohol abuse or smoking; 5) history of administering bisphosphonates; 6) history of bone metabolism disorder; and 7) history of cardiovascular disease.

The enrolled patients were divided into two groups according to the grafts used during surgery. Patients in group I underwent grafting with autogenous bone, and patients in group II underwent grafting with a composite of BMP2-CPC and autogenous bone at a ratio of 1:1 by volume. Radiographic evaluation was performed by analysing cone-beam computed tomographic (CBCT) data, and histological evaluation was performed by hematoxylin-eosin (HE) and Masson staining.

### Surgical procedures

After general anaesthesia, autogenous bone harvesting and cleft dissection were conducted simultaneously in a two-team approach. A line was marked above the anterior superior iliac spine. In group I, an incision with a mean length of 6 cm through the subcutaneous fat and muscle was made along the marked line. After exposure, the autogenous bone was harvested by the traditional trap door technique. In group II, minimally invasive iliac bone harvesting was performed within an improved incision with a mean length of 2 cm. After the small circular iliac cortex was cored out, trabecular bone cores were harvested using an osteotome. The surgical windows of both techniques were closed by a layered suture after reattaching the periosteum.

**FIGURE 1:**
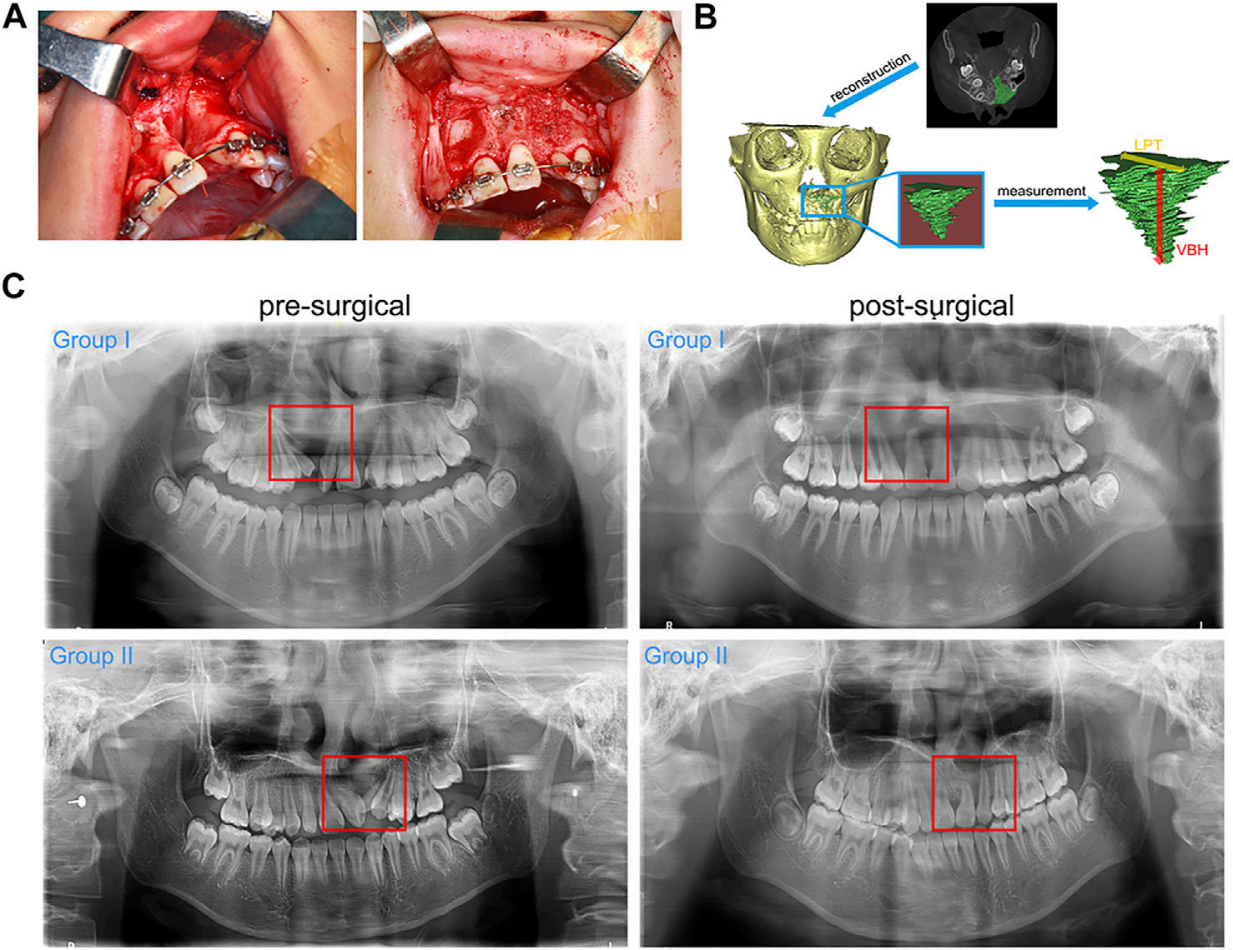
ABG surgery and radiographic examination. **(A)**. The key steps of ABG surgery: preparation of graft bed and graft filling; **(B)**. Three-dimensional reconstruction of the grafted area and related radiographic measurement; **(C)**. Representative pre-surgical and post-surgical (1 year after ABG) panoramic radiographs of each group (red box marks the alveolar cleft sites).

Simultaneously, the oral cavity was cleaned with 0.1% chlorhexidine gluconate solution and the gingiva and upper buccal sulcus were infiltrated with primacaine adrenaline. An anterior incision through the periosteum was placed in the gingival sulcus of the two adjacent incisors and continued on the ridge between the labial and palatal gingiva. A lateral incision was made along the edges of the cleft, and mucoperiosteal flaps were then raised to completely expose the surgical area. The oronasal fistula was closed by reconstruction of the nasal floor. Everting mattress sutures were used to suture palatal mucoperiosteal flaps together. The alveolar cleft cavity was filled with the autogenous bone graft or composite graft mentioned in the section of Study design (Figure 1A). Labial gingival flaps were sutured with absorbable 5–0 sutures (Vicryl, Ethicon, United States) in a tension-free manner. Post-surgical broad-spectrum antibiotics were routinely prescribed for at least 3 days, and 0.12% chlorhexidine gluconate oral rinses were used twice daily for oral hygiene maintenance.

### General evaluation

General evaluation was performed according to the clinical parameters (surgical duration, length of stay, wound healing and post-surgical complications) and panoramic radiographs. Standardized digital panoramic radiographs obtained before surgery and at 1 year after surgery were retrieved for evaluation of bone healing and canine eruption at the grafted sites. Clinically successful alveolar bone regeneration was defined as radiopaque bony bridge at the grafted sites.

### Radiographic evaluation

Routine CBCT examinations were performed immediately after surgery and at 3 and 6 months after surgery. The obtained CBCT data were processed and transformed into three-dimensional views using Simplant Pro Software (Materialise, Belgium). The bony contour of grafted areas was outlined in transverse CBCT images and was then stacked into three-dimensional models. Parameters related to the grafted areas were measured as follows: 1) volume (V), 2) vertical bone height (VBH), and 3) labiopalatal thickness (LPT). VBH was defined as the distance from the top level to the bottom level of the grafted areas. LPT was defined as the distance from the labial boundary to the palatal boundary of the grafted areas (Figure 1B). In addition, the three parameters were analysed in terms of resorption value and rate, as illustrated in Table.1.

**TABLE 1:** Analysis of radiographic parameters.

	Volume	VBH	LPT
Resorption value	V_im_-V_p_	VBH_im_-VBH_p_	LPT_im_-LPT_p_
Resorption rate	(V_im_-V_p_)/V_im_x100%	(VBH_im_-VBH_p_)/VBH_im_x100%	(LPT_im_-LPT_p_)/LPT_im_x100%

Vim: immediate volume, Vp: post-surgical volume (3-months or 6-months); VBHim: immediate vertical bone height, VBHp: post-surgical vertical bone height (3-months or 6-months); LPTim: immediate labiopalatal thickness, LPTp: post-surgical labiopalatal thickness (3-months or 6-months).

### Histological evaluation

Of the 19 enrolled patients, 1 patient from group I and 2 patients from group II underwent surgically assisted rapid maxillary expansion surgery at 3 years after ABG. Bone samples obtained from the grafted sites during surgery were collected for histological examination. The harvested bone samples were fixed in 4% paraformaldehyde (PFA), decalcified in 10% ethylene diamine tetraacetic acid (EDTA), dehydrated in a gradual series of ethanol (70–100%), and embedded in paraffin for sectioning. Five-μm-thick sections were subjected to HE and Masson staining according to standard protocols. The stained sections were captured under an upright microscope (Nikon, Japan). The bone area was measured in five randomly selected fields of vision by a blind observer using ImageJ software (National Institutes of Health, United States). The relative area of bone tissue was calculated as the bone area divided by corresponding total area of each vision.

### Statistical analysis

All quantitative measurements are presented as the mean ± standard deviation and were analysed using SPSS 22.0 (IBM, United States). For the general and radiographic evaluation, the Mann-Whitney test was used to detect differences. The data derived from histological examination were analysed by unpaired *t*-test to identify differences between group I and group II. The significance level was set at *(*p* < 0.05).

## Results

### General evaluation

Among the enrolled patients, 9 patients with a mean age of 10.89 ± 1.45 years were included in group I, and 10 patients with a mean age of 10.40 ± 1.28 years were included in group II. All patients healed well post-surgically, and no major post-surgical complications were recorded. Only one patient in group II encountered soft tissue swelling in the grafted sites. This symptom peaked 3 days after ABG and then receded completely with no wound dehiscence or graft exposure reported. No patients in either group required regrafting. Analysis of clinical parameters indicated no significant difference between group I and group II (Table.2). Pre-surgical panoramic radiographs showed unilateral discontinuity in the maxillary anterior region. Impacted canines or missing teeth could be observed on the cleft side of the maxillary anterior region. The post-surgical panoramic radiographs obtained at 1 year after ABG showed bony bridge filling in the cleft sites of all patients. Both the autogenous bone graft and the composite graft achieved satisfactory bone healing at the grafted sites and were well incorporated into the maxillary alveolar bone after 1 year of bone remodeling. The movement of canines towards the grafted sites was observed in both groups, and no sign of impeded canine eruption was recorded in either group (Figure 1C).

**TABLE 2:** Evaluation of clinical parameters.

	Group I	Group II	*p* value
Numer of patients	9	10	-
Ages (y)	10.89 ± 1.45	10.40 ± 1.28	0.497
Surgical duration (h)	2.49 ± 0.23	2.44 ± 0.18	0.661
Length of stay (d)	7.78 ± 1.03	7.60 ± 0.66	0.720
Wound healing	Normal	One case with soft tissue swelling in the grafted sites	-
Post-surgical complications	None	None	-
Impacted canines (cases)	4	5	-
Missing teeth (cases)	3	3	-

### Radiographic evaluation

**FIGURE 2:**
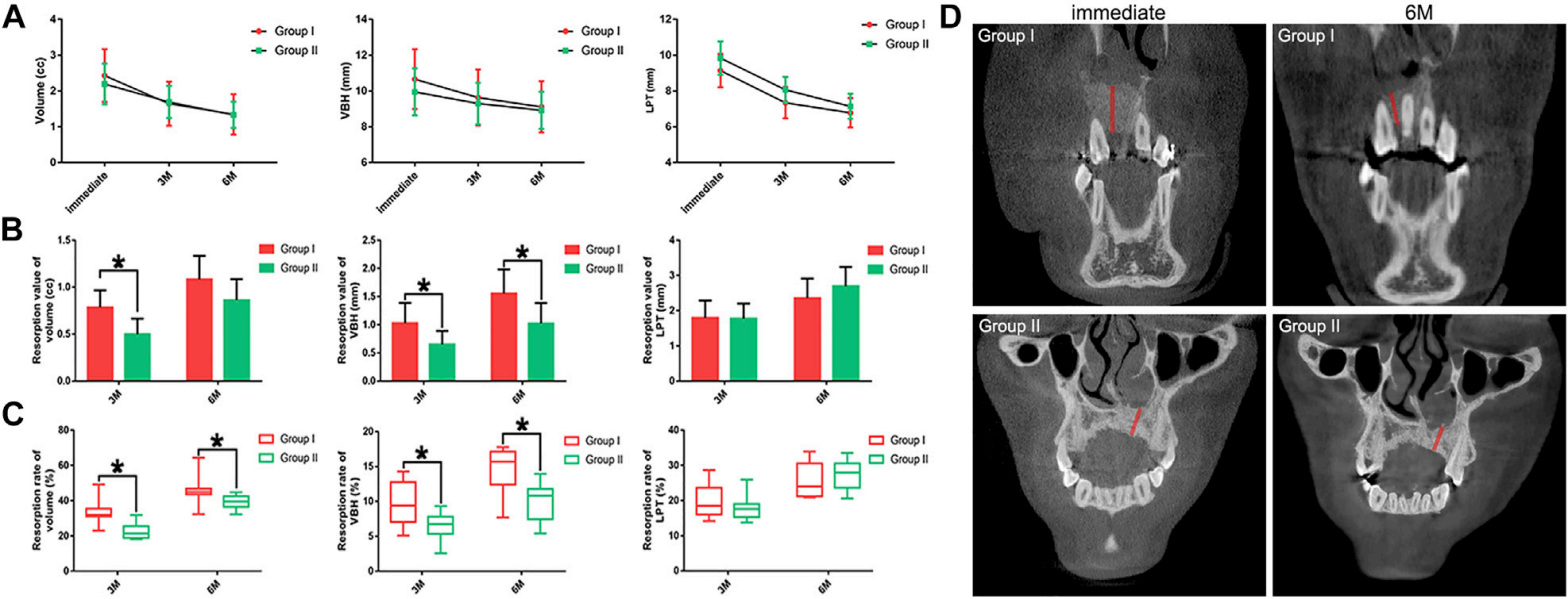
Radiographic evaluation results. **(A)**. The post-surgical volume, VBH and LPT of each group; **(B)**. Resorption values of the post-surgical volume, VBH and LPT of each group; **(C)**. Resorption rates of the post-surgical volume, VBH and LPT of each group; **(D)**. Representative CBCT images of the alteration of VBH in each group (red line marks the bone height of the grafted areas).

The alteration patterns of the parameters related to the grafted areas indicated a decreasing trend in the post-surgical volume, VBH and LPT of both groups. No significant differences were detected in the three parameters between group I and group II (Figure 2A). Figure 2B demonstrates that the resorption values of volume and VBH of group I were higher than those of group II, while there were no significant differences in the resorption values of LPT between the two groups. Similarly, Figure 2C shows higher resorption rates of volume and VBH for group I, and no significant differences were detected in the resorption rates of LPT between group I and group II. The mean post-surgical resorption values and rates are listed in Table.3. The finding related to the accelerated resorption of autogenous bone graft was supported by the evidence derived from CBCT images, in which we observed an apparent reduction in the VBH of group I at 6 months after the surgery (Figure 2D).

**TABLE 3:** Radiographic evaluation results.

Parameters	Group I	Group II	*p* value
3-months resorption value of volume (cc)	0.79 ± 0.17	0.49 ± 0.16	0.001*
6-months resorption value of volume (cc)	1.08 ± 0.23	0.86 ± 0.21	0.156
3-months resorption rate of volume (%)	33.42 ± 6.54	22.52 ± 4.21	0.001*
6-months resorption rate of volume (%)	45.89 ± 7.75	39.42 ± 3.68	0.006*
3-months resorption value of VBH (mm)	1.03 ± 0.34	0.65 ± 0.23	0.028*
6-months resorption value of VBH (mm)	1.56 ± 0.40	1.03 ± 0.34	0.013*
3-months resorption rate of VBH (%)	9.65 ± 2.95	6.44 ± 1.78	0.022*
6-months resorption rate of VBH (%)	14.53 ± 3.09	10.09 ± 2.55	0.003*
3-months resorption value of LPT (mm)	1.80 ± 0.46	1.78 ± 0.40	0.905
6-months resorption value of LPT (mm)	2.36 ± 0.52	2.70 ± 0.52	0.156
3-months resorption rate of LPT (%)	19.68 ± 4.60	17.93 ± 3.27	0.549
6-months resorption rate of LPT (%)	25.77 ± 4.89	27.32 ± 4.02	0.400

### Histological evaluation

**FIGURE 3:**
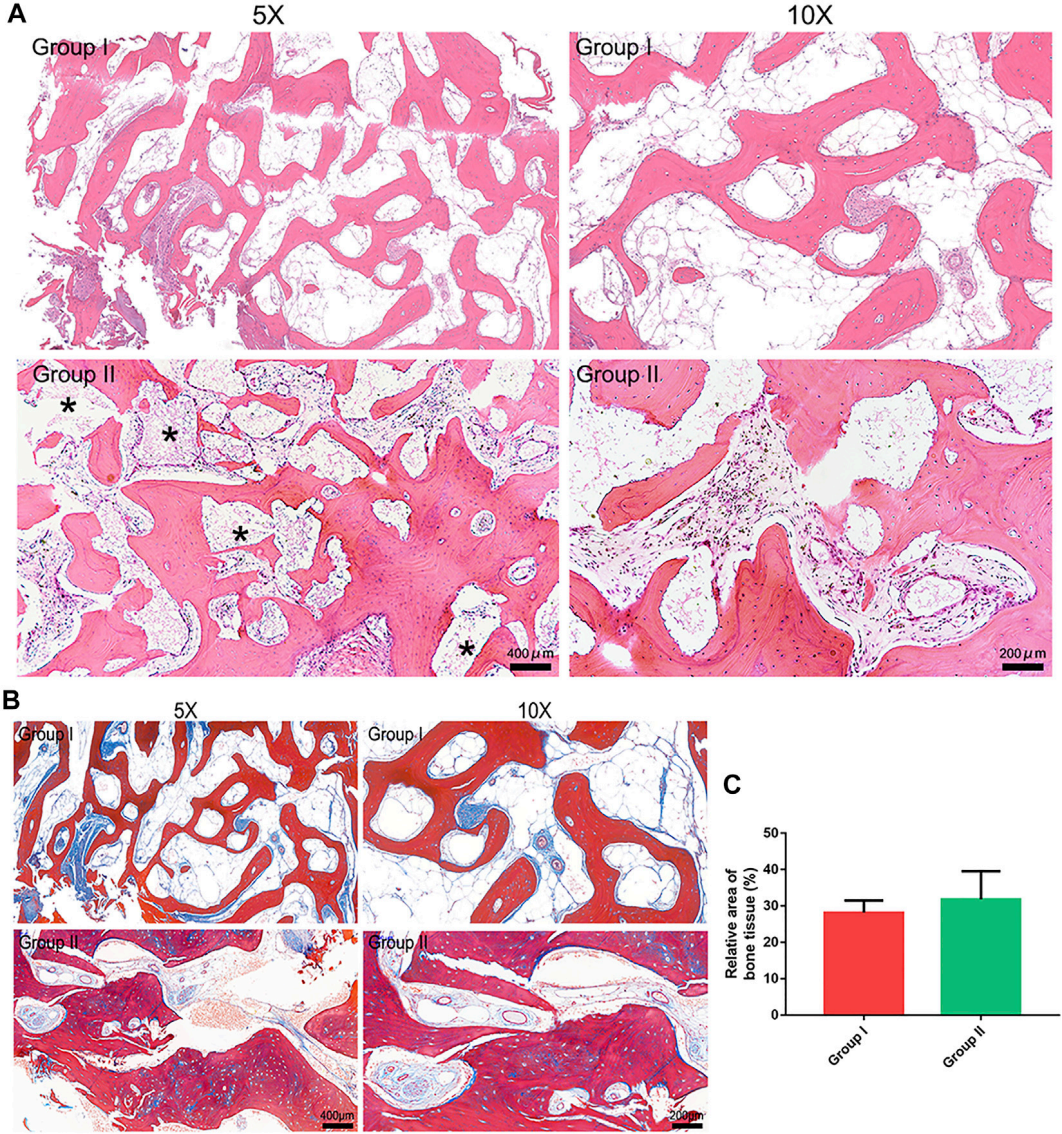
Histological evaluation results. **(A)**. HE staining results (* marks the residual BMP2-CPC particles); **(B)**. Masson staining results; **(C)**. Quantitative analysis of regenerated bone area in each group.

The long-term bone healing patterns at the grafted sites were examined by HE staining, and representative images are presented in Figure 3A. The grafts of both groups achieved a satisfactory bone healing outcome characterized by mature bone structure comprising trabeculae and bone marrow. The Masson staining results indicated that the mature level of bone in group II was similar to that in group I (Figure 3B). Both grafts underwent a resorption process and were fully or partly replaced by regenerated bone. Notably, the autogenous bone graft in group I were fully resorbed, while a few BMP2-CPC particles remained at the grafted sites in group II. This finding corroborated with the radiographic result that the composite graft were better than the autogenous bone graft in preserving the post-surgical bony contour. Quantification of regenerated bone area revealed that both the autogenous bone graft and the composite graft facilitated evident bone regeneration at the cleft sites (Figure 3C). Although quantitative analysis indicated no significant difference between the two grafts, the histological result showed thicker trabecular bone induced by the composite graft.

## Discussion

Secondary ABG, conducted in mixed dentition before canine eruption, is now regarded as a standard treatment for patients with alveolar cleft (Fahradyan et al., 2019; Mundra et al., 2022). Although autogenous bone grafting has remained the mainstream, its main drawbacks, donor site morbidity and limited bone supply, prompt clinicians to develop alternative materials that could meet the complicated clinical requirements of ABG (Kang, 2017; Francisco et al., 2021; Dissaux et al., 2022). Therefore, the appeal of BMP2 as a potential alternative is to avoid autogenous bone harvesting while preserving the osteogenic potential of the graft (da Rosa et al., 2019; Motamedian et al., 2022; Chou et al., 2022). In a recent umbrella review, Francisco et al. (Francisco et al., 2021) reported that the use of BMP2 produced similar results regarding bone volume, filling and height to the standard technique of autogenous bone grafting. To compensate for deficiencies in the carrier of BMP2, some researchers recommended a combined use of BMP2 and DBB to remedy the inability of ACS to resist compressive forces within the alveolar cleft sites (Francis et al., 2013; Hammoudeh et al., 2017; Liang et al., 2017; Mehta et al., 2018). The studies of Liang et al. (Liang et al., 2017) and Hammoudeh et al. (Hammoudeh et al., 2017) demonstrated that the combined use of BMP2 and DBB was equivalent to iliac crest bone grafting at 6–9 months post-surgically in terms of the percentage of bone ingrowth within the alveolar cleft sites and overall regenerated bone density, and the rates of major complications were similar between the two grafts. Nevertheless, the application of DBB only mitigates the drawback of BMP2-ACS in mechanical properties, but the issues regarding the release kinetics of BMP2 and the inherent drawback of DBB in potential disease transmission remain unaddressed (Liang et al., 2018; da Rosa et al., 2019; Liang et al., 2017; Um et al., 2020).

Unlike the simple combination of BMP2 and DBB, the porous structure of BMP2-CPC could gently immobilize BMP2 and effectively maintain the secondary structure of BMP2(18). The release of BMP2 exhibited a two-stage kinetic profile: an initial rapid release in the first 24 h followed by sustained slow release (Lin et al., 2016). These release kinetics could rapidly establish local accumulation of BMP2 at the early stage of bone healing and maintain the effective concentration of BMP2 for a long time. As a result, BMP2-CPC achieved satisfactory bone-repairing outcomes in both experimental rabbit distal femur defect models and the clinical treatment of long bone fractures (Lin et al., 2016). In addition to the improved release kinetics, the enhanced osteogenic capability of BMP2-CPC allowed for its successful application in the clinical practice of guided bone regeneration (Shen et al., 2021a). This evidence was in agreement with the present finding that the composite graft comprising BMP2-CPC and autogenous bone was similar to the autogenous bone graft in repairing alveolar clefts, and noticeably, few post-surgical complications were recorded in the composite graft group. To date, the most controversial argument against the application of BMP2 in the treatment of alveolar cleft is the increased rates of soft tissue swelling in the grafted sites (da Rosa et al., 2019; Hammoudeh et al., 2017). Mannion et al. (Mannion et al., 2011) suggested that many of the post-surgical complications of BMP2 were dose-related, and low-dose use of BMP2 was sufficient to promote good bone healing while producing fewer BMP2-related complications. Thus, this strategy of composite grafting with BMP2-CPC and autogenous bone was adopted to reduce the dosage of BMP2. According to the recommendations of previous studies, the mixing ratio of BMP2-CPC and autogenous bone was set as 1:1 by volume (Pripatnanont et al., 2009; Thuaksuban et al., 2010; Aly and Hammouda, 2016). Consequently, the present rate (10%) of soft tissue swelling was lower than the documented rate (14%) caused by total grafting with BMP2-loaded DBB(25). Generally, almost all of the cases complicated by BMP2-stimulated soft tissue swelling were self-limited and resolved at 3 or 4 days after surgery without further sequelae (da Rosa et al., 2019; Liang et al., 2017; Hammoudeh et al., 2017). Notably, it is worth mentioning that the amount of harvested bone was reduced 50% in group II by using the composite strategy. Although the present evaluation of clinical parameters indicated no significant differences between the traditional iliac bone harvesting and the minimally invasive technique, a previous review reported that, in the large-sample clinical studies, the minimally invasive technique achieved better clinical performance in terms of length of stay, donor site wound healing and post-surgical pain than the traditional method did (Saha et al., 2019).

To evaluate the bone healing after ABG, quantitative measurements were performed on CBCT images in this study, which yielded results different from those obtained from traditional two-dimensional radiographs. The two-dimensional examination methods, including occlusal, periapical and panoramic radiographs, were questioned in terms of limited accuracy and reproducibility resulting from image distortion, enlargement and overlap (Meazzini et al., 2016). Unlike two-dimensional radiographs, CBCT can produce sub-millimeter three-dimensional reconstruction models of alveolar clefts possessing higher precision (Liang et al., 2017). In agreement with the documented shrinking trend in the bony contour after ABG (Feichtinger et al., 2007; Feichtinger et al., 2008), the present radiographic results indicated that the decreasing trends in the post-surgical volume, VBH and LPT were coincident in group I and group II. The results of LPT resorption indicated no difference between group I and group II, which might be attributed to the mechanical pressure from the overlying labial and palatal soft tissue. Within this local stressful environment, BMP2 was capable of enhancing osteoclastogenesis by up-regulating the expression of nuclear factor-κ B ligand and down-regulating the expression of osteoprotegerin in osteoblasts (Kim et al., 2014; Kim et al., 2020), leading to the horizontal resorption of the bony contour. Moreover, horizontal soft tissue pressure could result in the dislocation of grafting materials during the bone healing period and compromise the stability of the bony contour at the labiopalatal level (Jiang et al., 2018). Although the advantage of the composite graft was inapparent in the evaluation of LPT, the evaluation of volume and VBH highlighted the merit of applying BMP2-CPC in the restoration of alveolar clefts. Compared to the autogenous bone graft, the composite graft had improvement in resistance to the resorption of the post-surgical bone volume and VBH. A previous review on the clinical performance of DBB suggested that DBB was better than autogenous bone in preserving the reconstructed bony contour (Baldini et al., 2011). Our previous study demonstrated that BMP2-CPC was similar to DBB in preserving the bone volume and height over the 6-months follow-up after guided bone regeneration (Shen et al., 2021a). Thus, with the addition of BMP2-CPC, the composite graft exhibited improved performance in preserving post-surgical bone volume and VBH, which is beneficial to subsequent implantation and orthodontic treatments.

Notably, since the CPC scaffold is a radiopaque material that will preclude accurate visualization of regenerated bone within the alveolar cleft sites, we further conducted histological examination to observe the pattern of long-term bone healing after ABG and quantify the bone formation-related parameters at the microscopic level. The present histological results indicated that BMP2-CPC was an eligible alternative material for application in ABG, as was evidenced by the well-organized bone structure in the composite graft-filled cleft sites and the considerable amount of regenerated bone comparable to the amount of autogenous bone graft-induced bone. The favourable osteogenic phenomenon induced by the composite graft relies on the presence of BMP2-CPC and autogenous bone, both of which are potent osteoconductive and osteoinductive grafts (Aghali, 2021). Our previous study revealed that BMP2-CPC could induce a robust pattern of bone regeneration through both direct stimulation with BMP2 and indirect immunoregulatory effect (Shen et al., 2021b). More importantly, BMP2-CPC possessed not only enhanced osteogenic properties, but also favourable resorption performance. As coincident with the radiographic finding that the resorption rate of BMP2-CPC was slower than that of autogenous bone, the histological results showed a small number of residual BMP2-CPC particles remaining at 3 years after surgery. Nevertheless, compared to DBB, which is an inert material that can remain sequestered in local bone tissue for up to 10 years (Sartori et al., 2003; Baldini et al., 2011), BMP2-CPC seemed to be a better choice according to its accelerated resorption rate (Shen et al., 2021a). In addition, the finding derived from panoramic radiographs that BMP2-CPC would not increase the risk of impeded bone remodeling and canine eruption further supported the use of the composite graft.

Despite the nuanced understanding of bone regeneration at the alveolar cleft sites that this study has provided, several questions remain unaddressed. The present results should be interpreted with caution due to the small number of subjects and the short-term follow-up of radiographic evaluation. Considering the present limited control group, the strength of evidence would be bolstered if a BMP2-CPC-grafting control group and a control group that underwent grafting with autogenous bone harvested by minimally invasive technique could be added. Although the present study achieved quantitative evaluation through CBCT examination, the boundaries of the original bone defect at multiple time points were hard to be exactly identified in a reproducible manner. Given these limitations, a randomized controlled clinical trial with large samples, comprehensive experimental designs and long-term follow-up should be performed in the future studies. Histological examination of bone samples at 3 and 6 months after ABG is needed to reveal the differences in short-term bone healing at the alveolar cleft sites between the composite graft and the autogenous bone graft. Moreover, whether the use of BMP2-CPC alone can be an alternative to autogenous bone graft for application in ABG and relevant issue on cost-effectiveness should be addressed in the future with sound support of prospective studies.

In conclusion, the present results demonstrated that the composite graft comprising BMP2-CPC and autogenous bone harvested by minimally invasive technique exhibited satisfactory bone-repairing efficacy and favourable resorption performance, making the composite graft an eligible choice for the clinical practice of ABG.

## Data Availability

The original contributions presented in the study are included in the article/supplementary material, further inquiries can be directed to the corresponding authors.
